# Secreted APP regulates the function of full-length APP in neurite outgrowth through interaction with integrin beta1

**DOI:** 10.1186/1749-8104-3-15

**Published:** 2008-06-23

**Authors:** Tracy L Young-Pearse, Allen C Chen, Rui Chang, Cesar Marquez, Dennis J Selkoe

**Affiliations:** 1 Center for Neurologic Diseases, Brigham and Women's Hospital and Harvard Medical School, Boston, MA 02115, USA

## Abstract

**Background:**

β-Amyloid precursor protein (APP) has been reported to play a role in the outgrowth of neurites from cultured neurons. Both cell-surface APP and its soluble, ectodomain cleavage product (APPs-α) have been implicated in regulating the length and branching of neurites in a variety of assays, but the mechanism by which APP performs this function is not understood.

**Results:**

Here, we report that APP is required for proper neurite outgrowth in a cell autonomous manner, both *in vitro *and *in vivo*. Neurons that lack APP undergo elongation of their longest neurite. Deletion of APLP1 or APLP2, homologues of APP, likewise stimulates neurite lengthening. Intriguingly, wild-type neurons exposed to APPs-α, the principal cleavage product of APP, also undergo neurite elongation. However, APPs-α is unable to stimulate neurite elongation in the absence of cellular APP expression. The outgrowth-enhancing effects of both APPs-α and the deletion of APP are inhibited by blocking antibodies to Integrin β1 (Itgβ1). Moreover, full length APP interacts biochemically with Itgβ1, and APPs-α can interfere with this binding.

**Conclusion:**

Our findings indicate that APPs-α regulates the function of APP in neurite outgrowth via the novel mechanism of competing with the binding of APP to Itgβ1.

## Background

β-Amyloid precursor protein (APP) is a type 1 membrane glycoprotein found at the cell surface and in endosomal, endoplasmic reticulum, and Golgi membranes in many cell types [1-3]. APP is expressed in the developing and mature brain at the somal cell surface, along growing axons and at the axonal growth cone. Cleavage by β- and then γ-secretase generates Aβ peptide, the apparent pathogenic agent in Alzheimer's disease (reviewed in [4]). However, during brain development, a much larger fraction of APP is processed by α-secretases, which cleave within the Aβ sequence [5]. This cleavage releases a soluble, amino-terminal APP fragment (referred to as APPs-α) and leaves a membrane-retained carboxy-terminal fragment (CTF) of 83 amino acids (C83). γ-Secretase can then cleave C83, yielding a free intracellular domain (AICD) and a small secreted peptide termed p3 [6].

APP has two homologues in mouse, human and other mammals, APLP1 and APLP2 [7,8], which also may be processed by α-, β, and γ-secretases in a manner similar to APP [9-13]. Targeted deletions of these genes reveal functional redundancy among the three APP family members. Single genetic deletions in APP, APLP1 or APLP2 are each viable, whereas APLP2 deletion in combination with either APP or APLP1 deletion leads to early postnatal lethality [14-16]. The *in vivo *effects of disruption of these genes in mice have been difficult to pinpoint due to this functional redundancy. However, cells from these mice can be cultured and manipulated *in vitro *to examine the phenotypic effects of these deletions in detail.

Neuronal and glial co-cultures plated from the hippocampi of APP knock-out mice displayed shorter axonal outgrowth after 1 day *in vitro *(DIV) and longer axons after 3 DIV compared to neurons plated from wild-type brains [17]. These results suggested that deletion of endogenous APP initially inhibits neurite outgrowth but over time stimulates neurite outgrowth, supporting the hypothesis that APP may first regulate adhesion to the substratum and then act to inhibit neurite outgrowth.

The secreted extracellular domain of APP, which is endogenously produced by α-secretase (APPs-α) or β-secretase (APPs-β), has also been shown to play functional roles in several assays. Although APPs-α can rescue some of the effects of APP deletion [18], studies of neurite outgrowth in culture have not directly tested whether APPs-α application can rescue the effects of APP deletion. Although neuronal and glial co-cultures from APP knock-out mouse hippocampi displayed longer axons after 3 DIV than neurons from wild-type brains [17], these authors found that co-culture of wild-type or APP knock-out neurons with APP knock-out astrocytes increased neurite outgrowth [17]. The latter result taken by itself suggests that APPs secreted by astrocytes would normally inhibit neurite outgrowth. In contrast, other studies have shown that exogenous addition of APPs-α to cultured neurons actually promotes neurite outgrowth in a fashion similar to deleting APP in neural cultures. For example, APPs-α was found to increase neurite outgrowth in several assays, including in primary cortical cells [19], cortical explants [20], and PC12 cells [21,22]. Interpretation of these various results requires a more complex explanation for how full length APP and soluble APPs-α from neurons and glia interact functionally.

Several lines of evidence suggest that APP may interact functionally with integrins to mediate neurite outgrowth. Similar to APP, integrins have been shown to regulate neurite outgrowth [23,24], and APP and integrins (α5, α1, and β1) co-localize in developing neurons in both growth cones and along axons [25-27]. However, no studies have addressed whether APP and integrin family members functionally or biochemically interact.

As reviewed above, both the absence of APP and the application of its major secreted derivative (APPs-α) can elongate neurites in culture. These seemingly paradoxical observations, which we confirm herein, led us to hypothesize that APPs-α may act to oppose effects of cell-surface APP and its homologues by competitively binding to factors that normally interact with the holoprotein to inhibit neurite outgrowth. Here, we show that deletion of each of the full-length APP family members acts in a cell-autonomous manner to promote neurite outgrowth in primary neuronal cells, and that adding the soluble derivative from each APP family member to wild-type cells similarly stimulates neurite outgrowth. Importantly, we show that APPs-α does not stimulate neurite outgrowth in the absence of APP. Our results support the hypothesis that secreted APPs-α can inhibit the activity of cell-surface APP. Further, we provide evidence that APP and APPs-α can each interact biochemically and functionally with integrins to mediate these effects.

## Results

**Figure 1:**
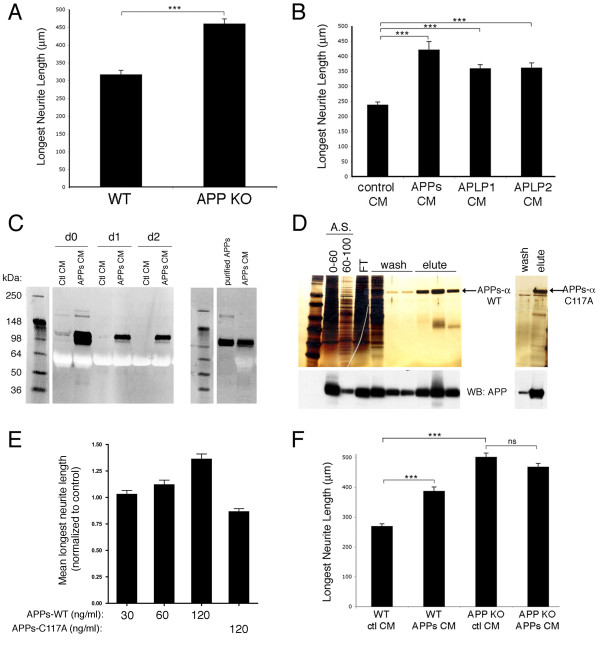
**Loss of APP and APPs-α application each increase neurite length**. E18 primary hippocampal or cortical neurons from wild-type (WT) or APP knock-out (APP KO) mice were treated as described. Three days later, the cells were fixed and immunostained for βIII-tubulin, and neurite length was measured. **(a) **Comparison of neurite lengths of wild type and APP knock-out hippocampal neurons. **(b) **Comparison of neurite lengths of wild-type hippocampal neurons treated with CM from untransfected, APPs, APLP1, or APLP2 expressing CHO cells. **(c) **Western blots for APPs (8E5) on CM from day 0 (d0), d1, or d2 after addition to primary neurons (left panel) or western blot of equal amounts of purified APPs-α compared to APPs-α conditioned media (right panel). **(d) **Silver stain of sequential purification steps of APPs-α from baculovirus-transduced insect cells. Left panel: lanes 1 and 2 are ammonium sulfate (A.S.) precipitations from 0–60% or 60–100%; lane 3 is the flow through (FT) after addition to a nickel chelating column; lanes 4–6 are washes from the column; and lanes 7–9 are elutes from column. Right panel: final purified product of APPs-α with mutation of cysteine 117 (see Materials and methods for details of purification). Bottom: western blot (WB) with an antibody that recognizes the extracellular domain of APP (8E5, Elan) **(e) **Quantification of neurite length after addition of purified APPs-α (wild type) at increasing concentrations or APPs-α (C117A) at 120 ng/ml. **(f) **E18 primary cortical neurons from APP knock-out or wild-type littermates were treated with CM from either CHO cells stably expressing APPs-α or control CHO cells. Three days later, cells were fixed and immunostained for βIII-tubulin, and neurite length was measured. Error bars represent standard error of the mean; *p < 0.05; ***p < 0.001.

In order to confirm and extend previous studies showing that APP plays a role in neurite outgrowth, we plated embryonic day 18 (E18) primary hippocampal or cortical neurons from APP knock out and wild-type littermate mice on CC2 coated two-chamber slides. After 3 DIV, cells were fixed and immunostained for βIII-tubulin, and the length of the longest neurite (defined here as the axon) was measured. Using these culture conditions, APP knock out cultures displayed longer axons than did neurons in wild-type cultures for both hippocampal (Figure 1a) and cortical (Figure 1f) neurons.

Next, we sought to determine the effects of APPs-α in this neurite outgrowth assay. Conditioned media (CM) from either Chinese hamster ovary (CHO) cells stably expressing human APPs-α or untransfected (control) CHO cells were added to wild-type E18 primary neurons for three days. APPs-α in the CM was present over the 3-day period after some degradation during the first 24 h (Figure 1c). In agreement with previous studies, the CM of the APP-overexpressing cells increased axon outgrowth over three days (Figure 1b). We found that the CM of CHO lines stably expressing APLP1 or APLP2 [10] conferred similar axon-elongating activity (Figure 1b).

Besides APPs-α, whole CM from the CHO transfectants contains numerous additional proteins that could act in conjunction with or oppose the neurite-elongating activity of APPs-α. To assess whether APPs-α by itself can induce axonal outgrowth, His-tagged APPs-α was purified from baculovirus-transduced insect cells. Pure, recombinant APPs-α induced axonal outgrowth to a level similar to APPs-α from CM when similar concentrations of APPs-α were applied (Figures 1c–e). In contrast, purified APPs-α with a mutation of cysteine 117, which is required to form a disulfide bridge important for determining the secondary structure of the extracellular domain of APP [28], did not increase the length of neurites in this assay (Figure 1d, e).

Because both loss of APP and addition of APPs-α induced an increase in axonal outgrowth in this assay, we hypothesized that APPs-α may bind to factors that the extracellular domain of cell-surface APP also binds to, thereby acting to compete with cell-surface APP and inhibit its neurite-regulating activity. As an initial test of this hypothesis, we applied APPs-α to APP knock-out neurons to investigate whether APPs-α would still increase axonal outgrowth in the absence of cell surface APP. Although APPs-α again increased axonal outgrowth in wild-type neurons, it did not further increase outgrowth in neurons plated from APP knock-out littermates (Figure 1f). This result indicates that APP must be present for the neurite-enhancing effects of APPs-α to occur.

**Figure 2:**
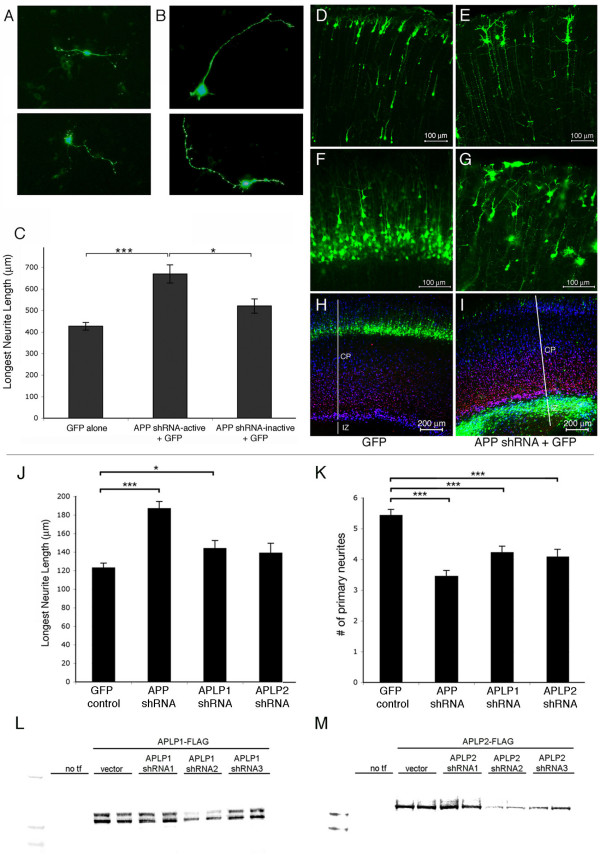
**APP knock-down increases neurite length in a cell-autonomous manner**. **(a, b) **E17 primary cortical neurons were plated and transfected with plasmids encoding GFP alone (a) or GFP with APP shRNA-active (b) or with APP shRNA-inactive (not shown). Three days later, neurons were fixed and immunostained for βIII-tubulin. **(c) **Neurite length was quantified in GFP+, transfected cells. Error bars represent standard error of the mean; *p < 0.05; ***p < 0.001. **(d-i) **E17 (d, e) or E14 (f-i) cortices were electroporated with GFP (d, f, h) or GFP + APP shRNA-active (e, g, i) and harvested at postnatal day 5. Images in (d-g) are of the upper half of the cortical plate in coronal sections. (h, i) Coronal sections immunostained for Tbr1 (red), marking layer VI of the cortical plate (CP), and MAP2 (blue), marking the entire cortical plate. GFP positive, electroporated regions of E14 cortices were dissected 48 h after electroporation, dissociated, and plated. Three days later cells were fixed and immunostained for βIII-tubulin. IZ; intermediate zone; white lines delineates noted regions of the cortex **(j, k) **Neurite lengths (j) or the number of primary neurites (k) were quantified for GFP+ cells. Error bars represent standard error of the mean; *p < 0.05; ***p < 0.001. **(l, m) **FLAG-tagged murine APLP1 (l) or APLP2 (m) constructs were co-transfected into CHO cells with an empty vector or with three different shRNA constructs targeting rodent APLPs. Transfections (tf) of duplicate wells are shown. Forty-eight hours post-transfection, cells were lysed, and the western blots for FLAG on protein normalized samples are shown.

When APP knock-out neurons are plated and examined for effects on neurite outgrowth as above, the cultures lack both full-length APP and secreted APPs-α. To determine whether APP is required in a cell-autonomous manner for neurite regulation, we transfected APP small hairpin RNAs (shRNAs) into primary rat neurons at a low efficiency. We purposefully achieved less than 1% transfection efficiency in primary neurons using a modified lipofectamine protocol, in order to examine cell autonomous effects. Several APP shRNAs were designed to target rodent APP, and two shRNAs were selected that either efficiently knocked down APP (APP-active) or did not knock it down (APP-inactive) [29]. E17 primary rat neurons were transfected with active or inactive APP shRNA plus green fluorescent protein (GFP) or else with GFP alone, and three days later, the cells were fixed and immunostained for βIII-tubulin. The transfected neurons were identified by their GFP fluorescence (Figure 2a, b), and their longest neurites were measured as before. Transfection of APP shRNA-active significantly increased the length of the longest neurite relative to transfection with either APP shRNA-inactive or GFP alone (Figure 2c), consistent with a cell-autonomous function of APP in regulating neurite length.

Next, we sought to determine whether the effects on neurites of altering APP expression that we observed in cultured neurons also occurred *in vivo*. To this end, control DNA (GFP alone) or APP shRNA-active plus GFP were electroporated *in utero *into E14 or E17 neuronal precursor cells lining the lateral ventricle of rat embryos. Data from our lab and others have shown that the rate of co-electroporation of two plasmids of equal molarity is between 90% and 95%, and that *in vivo *electroporation of APP shRNA and GFP results in an efficient knock down of APP in GFP-positive, electroporated cells [29-31]. The electroporated embryos were harvested at postnatal day 5, and their brains were fixed and sectioned coronally. With electroporation of GFP alone, neuronal precursors migrated into the cortical plate and differentiated, displaying radially aligned neurites, as expected (Figure 2d, f). Previously, we reported that co-electroporation of APP shRNA-active and GFP resulted in a strong decrease in APP expression and a corresponding defect in entry into the cortical plate for the large majority of developing neurons electroporated at E13 and harvested at E19, with greater than 80% of cells failing to migrate correctly [29]. Here, we electroporated APP shRNA constructs at E14 and harvested at a postnatal time point (postnatal day 5) when migration and differentiation are complete. The APP-silenced cells continued to show a defect in cortical plate entry at this late time point (Figure 2h versus 2i), and the cells that were able to migrate into the cortical plate exhibited abnormal neurites, with radial and tangential processes showing excessive branching and often a beaded appearance in subsets of cells (Figure 2e, g).

The phenotype described above following knock down of APP *in vivo *occurred in only a subset of cells and was difficult to quantify accurately. Therefore, in order to quantify the qualitative phenotype we observed *in vivo*, regions of the cortex electroporated at E14 were dissected two days after electroporation (to allow time for APP knock down) and cultured and analyzed using the same neurite outgrowth assay described above. Similar to the *in vitro *Lipofectamine transfection experiments, plating of the *in utero *electroporated cells revealed APP knockdown in only a small subset of all of the neurons (less than 10%), and GFP fluorescence was used to identify these electroporated cells. In agreement with the Lipofectamine transfection experiments, cells electroporated *in vivo *with APP shRNA-active displayed longer axons than those electroporated with GFP alone (Figure 2j). In addition, these cells showed fewer primary neurites (Figure 2k), but there was no significant difference in axonal branching between control GFP electroporations and APP shRNA-active plus GFP co-electroporations (data not shown).

APP has two family members (APLP1 and APLP2) that appear to play redundant roles with APP for at least some of its functions and may compensate for a loss of APP in knock-out mice [14-16]. As shown in Figure 1b, APLP1-s and APLP2-s application each induced an increase in axonal outgrowth. In order to determine whether knock down of APLP1 or APLP2 resulted in a similar increase in axonal outgrowth, shRNAs were generated that efficiently knock down each of these family members (Figure 2l, m) and electroporated *in utero *and analyzed as described for APP. These analyses showed a small but significant increase in axonal outgrowth upon APLP1 knock down (an increase with APLP2 knock down failed to reach significance) and significant decreases in the number of primary neurites with knock down of APLP1 or APLP2, relative to control GFP electroporations (Figure 2j, k).

**Figure 3:**
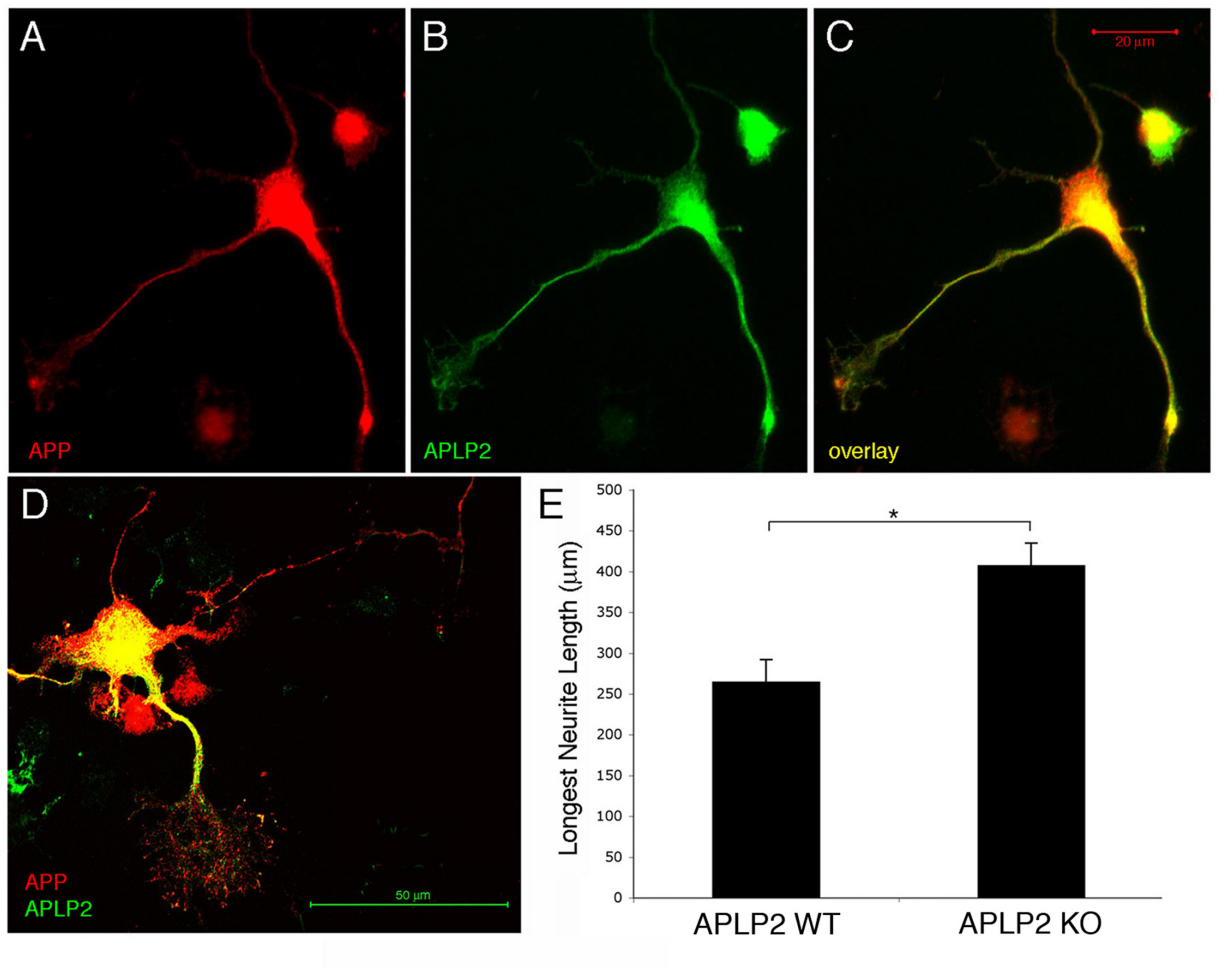
**APLP2 is expressed in primary neuronal processes and loss of APLP2 increases neurite length.** Primary E18 wild-type neurons were plated and fixed six days later. Neurons were immunostained for **(a, c, d) **APP and **(b, c, d) **APLP2. **(d, e) **E18 primary hippocampal neurons from wild-type (WT) or APLP2 knock out (KO) mice were plated. Three days later, cells were fixed and immunostained for βIII-tubulin and neurite length was measured and quantified. Error bars represent standard error of the mean; *p < 0.05.

Using this *in vivo *electroporation paradigm, the analysis is limited to the specific subset of cells that are electroporated (those that undergo their terminal mitosis on or near E14, the day of electroporation). Therefore, the lack of a significant change in axonal length with APLP2 knock down may be due to high endogenous expression levels of APLP2 in the subset of cortical cells electroporated at E14. To test whether APLP2 expression could be observed in embryonic cortical cells, APLP2 expression was analyzed in E18 primary cortical cultures. In these cultures, APP and APLP2 were expressed in similar patterns in cell bodies, neurites and growth cones in a subset of cells (Figure 3a–d). To test if APLP2 contributes to axonal outgrowth in such cells, primary neurons were plated from E18 APLP2 knock-out cortices. This analysis showed that deletion of APLP2 inall cortical cells resulted in a significant increase in axonal outgrowth (Figure 3e).

**Figure 4:**
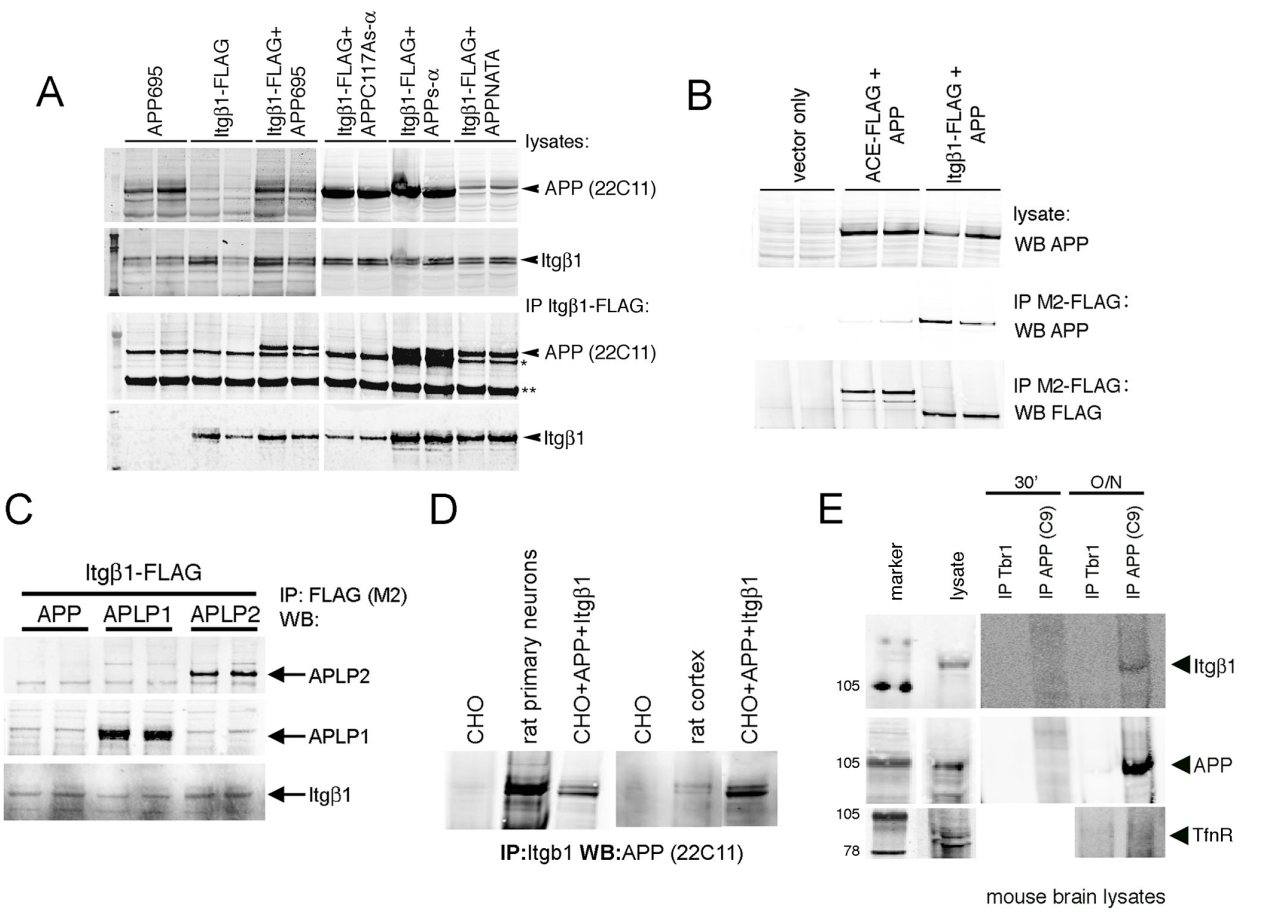
**APP and Itgβ1 biochemically interact**. **(a) **CHO cells were transiently transfected with constructs as shown. Co-immunoprecipitations of the resultant lysates were then performed with anti-FLAG agarose. Western blots for the amino terminus of APP (anti-APP; 22C11; Chemicon) or Itgβ1 (Cell Signaling): upper panels are western blots of lysates and lower panels show western blots of FLAG-immunoprecipitations (IP). The band denoted by the single asterisk is believed to be a background band. The band denoted by the double asterisks is the heavy chain of IgG. **(b) **CHO cells were transiently transfected with APP and Itgβ1-FLAG or another carboxy-terminally tagged type I-transmembrane domain protein, angiotensin converting enzyme (ACE). Co-immunoprecipitations of the resultant lysates were then performed with anti-FLAG agarose and western blotted (WB) for APP or FLAG, as shown. **(c) **CHO cells were transiently transfected with Itgβ1-FLAG and APP, APLP1, or APLP2. Co-immunoprecipitations of the resultant lysates were then performed with anti-FLAG agarose and western blotted for APLP1, APLP2, or Itgβ1, as shown. **(d) **Lysates from CHO cells, E18 rat primary neurons, rat cortex, or transfected CHO cells were immunoprecipitated for Itgβ1 and western blotted for APP. **(e) **Lysates from total mouse brain were immunoprecipitated with antibodies directed to APP or Tbr1 (used as a control) for 30 minutes or overnight (O/N) and western blotted for Itgβ1, APP, or transferrin receptor as a negative control.

Previous studies have shown that APP and integrins co-localize in primary neurons, specifically at points of substrate contact [25-27]. Similar to APP family members, integrins have been shown to regulate neurite outgrowth (for review, see [32]. In order to determine whether APP and integrins physically interact, CHO cells were transiently transfected with FLAG-tagged murine Integrin β1 (Itgβ1-FLAG) and the 695 amino acid splice variant of APP. The lysates of these cells were subjected to immunoprecipitation for Itgβ1 with M2 anti-FLAG antibody, and the immunoprecipitates were probed for APP, revealing the co-immunoprecipitation of APP and Itgβ1 (Figure 4a). The carboxy-terminal domains of both APP and Itgβ1 contain NPXY motifs, which have been shown to bind to common interacting partners such as Fe65 and Dab1 (for review, see [33,34]. To determine if this domain is necessary for APP-Itgβ1 co-immunoprecipitation, APP with two point mutations in this motif (APP-NATA) and the cleaved extracellular domain of APP (APPs-α) were tested for their ability to co-immunoprecipitate with Itgβ1. Both APP-NATA and APPs-α were able to interact with Itgβ1 (Figure 4a). In contrast, APPs-α with a point mutation in cysteine 117 did not co-immunoprecipitate with Itgβ1. Further, another type I transmembrane domain receptor (angiotensin converting enzyme) that was also FLAG-tagged at its carboxyl terminus and expressed at a level similar to APP did not co-immunoprecipitated with APP (Figure 4b). Similar to APP, APLP1 and APLP2 also co-immunoprecipitated with Itgβ1 (Figure 4c). These immunoprecipitation experiments were all performed with overexpressed proteins. In order to confirm that APP interacts with Itgβ1 under endogenous conditions in neuronal cells, co-immunoprecipitation experiments were performed on these proteins from mouse and rat brain homogenates, as well as in rat primary neurons, and these all confirmed the interaction (Figure 4d, e).

**Figure 5:**
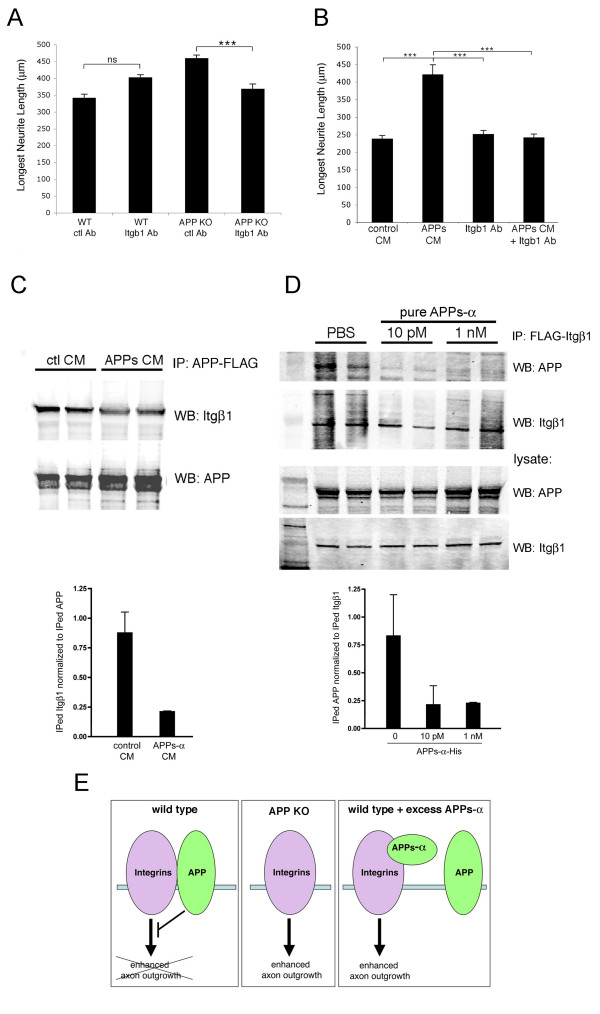
**Itgβ1 blocking antibody inhibits the effects on neurite outgrowth of APPs-α or deletion of APP**. **(a) **E18 primary cortical neurons from wild-type (WT) or APP knock-out (KO) mice were treated with Itgβ1 blocking antibody or control (ctl) antibody. **(b) **Wild-type E18 neurons were treated with control or APPs-rich CHO CM together with either Itgβ1 blocking or control antibody. The longest βIII-tubulin-positive neurites were quantified. Error bars represent standard error of the mean; ***p < 0.001; ns, not significant. **(c, d) **CHO cells were transfected with APP-FLAG and Itgβ1 (c) or Itgβ1-FLAG and APP (d). Example of western blot (WB) of co-immunoprecipitates of APP and Itgβ1 after incubation with control (ctl) CM or APPs-α CM (c) or purified His-tagged APPs-α (d) with quantification below; error bars represent standard deviation between duplicate wells. A significant decrease in co-immunoprecipitation between APP and Itgβ1 was observed between multiple experiments; *p *< 0.05. **(e) **Model for proposed functional interaction between APP and integrins in regulating neurite elongation.

To address whether APP and integrins interact functionally, E18 primary cortical neurons from wild-type or APP knock-out mice were plated and treated with an Itgβ1 blocking antibody (Ha2/5; BD Biosciences; San Jose, CA, USA) or an isotype-matched control antibody. Previous experiments have shown that blocking Itgβ1 function has been shown both to inhibit neurite outgrowth or to have no effect, based upon the assay utilized [24,35,36]. Our assay conditions did not reveal significant effects on neurite length of application of the Itgβ1 blocking antibody to wild-type neurons relative to the addition of the control antibody (Figure 5a, b). However, application of the Itgβ1 blocking antibody to APP knock-out neurons or else to APPs-α rich CM-treated cultures fully inhibited the axon-outgrowth promoting effect of each of these (Figure 5a, b).

Because APPs-α is able to biochemically interact with Itgβ1 in a fashion similar to full length APP (Figure 4a), we hypothesized that APPs-α may be able to compete with full-length APP for binding to Itgβ1. To address this possibility, APP with a carboxy-terminal FLAG tag (APP-FLAG) was co-transfected with Itgβ1 into CHO cells. After 48 h, CM from cells expressing APPs-α or from untransfected control cells was added to the co-transfected cultures for 8–12 h. Cultures were then lysed, and APP and Itgβ1 were co-immunoprecipitated with anti-FLAG agarose (Figure 5c). In a second approach, Itgβ1-FLAG was co-transfected with APP, and after 48 h, purified His-tagged APP was applied to the cultures for 8–12 h (Figure 5d). Quantification of these experiments using Licor imaging showed that in the presence of APPs-α, there was an approximately 70% decrease in the amount of APP-Itgβ1 co-immunoprecipitated relative to the amount of immunoprecipitated FLAG-tagged construct using both of these experimental approaches (Figure 5c, d). These results suggest that APPs-α can compete with full-length APP for binding to Itgβ1.

## Discussion

Studies from multiple labs have now established that APP plays a role in neurite outgrowth. However, the findings are sometimes inconsistent as to whether APP enhances or inhibits neurite outgrowth. It appears that APP may have differential effects on outgrowth and branching of neurites depending on the type of neuronal culture assayed (dissociated primary neurons, explants, or immortalized neuronal cell lines), the timing of analysis after plating (1 day versus 3 days versus 5 days), and the substrate upon which the neurons are plated (CC2, laminin, poly-D-lysine, fibronectin, and so on). In order to address the mechanism through which APP is acting to affect neurite outgrowth, we utilized a single primary neuronal culture system to directly compare the effects of genetic loss of APP and the application of APPs-α.

Loss of APP through either genomic deletion or shRNA-induced silencing resulted in significantly increased neurite elongation. Similarly, APPs-α application significantly stimulated outgrowth of the longest neurite in the same assay but, importantly, had no effect in cells that did not express APP. These results support our hypothesis that APPs-α acts to enhance neuronal process outgrowth by inhibiting the activity of cell surface APP, as diagrammed in Figure 5e. It should be noted that there are other possible explanations for the result; for example, the neuronal processes may not be competent to grow any longer beyond the length observed with APP deletion after 3 DIV. However, further support for the hypothesis derives from our experiments showing that APPs-α is able to block both the physical and functional interaction of full length APP and Itgβ1 (Figure 5). The model in Figure 5e incorporates these data to schematize the interactions we have shown to regulate neurite outgrowth in our assay. Under 'wild-type' conditions, APP inhibits the outgrowth-promoting activity of Itgβ1. In the absence of APP (that is, in APP knock out neurons or APP shRNA-transfected neurons), Itgβ1 enhances neurite outgrowth. Application of APPs-α to wild-type neurons can compete with cell surface APP for binding to Itgβ1 (as we demonstrate in Figure 5c, d), thus inhibiting the ability of APP to regulate Itgβ1-induced neurite outgrowth. In the absence of cell surface APP, APPs-α has no effect.

Like APP family members, integrins are single-pass, type 1 transmembrane proteins that mediate adhesion to extracellular matrix factors and regulate neurite outgrowth (reviewed in [37]). Integrins exist as heterodimers, and previous studies have shown that APP co-localizes with integrin α 1β1 and α 5β1 heterodimers in growth cones [25,26,38] and at point contacts but not focal adhesions in embryonic neurons [25]. Based upon data presented here, the extracellular domain of APP is able to interact with Itgβ1, and APPs-α can compete with full length APP for binding to Itgβ1 at the cell surface. Both APP and Itgβ1 have NPXY motifs that interact with Fe65, a cytoplasmic adaptor protein that has the ability to bind two NPXY motifs simultaneously to form tripartite complexes, as it does with APP and LRP [39,40]. However, full length APP with mutations of its NPXY domain retains the ability to interact with Itgβ1. Thus, APP is likely to interact with Itgβ1 through both its extracellular domain and indirectly through its intracellular domain via interactions with adaptor proteins such as Fe65. In agreement with a role for Fe65 in this process, Fe65 has been found to co-localize with APP and Itgβ1 in growth cones [26], and it was recently shown that blocking the ability of Fe65 to bind to APP slowed axonal outgrowth [41]. Our results showing a biochemical interaction between APP and Itgβ1 are in agreement with recent studies presented by the Rebeck lab [42].

The two other members of the mammalian APP family, APLP1 and APLP2, are also processed by α-, β-, and γ-secretases, releasing analogous cleavage products. The carboxy-terminal domains of all three family members are highly similar in amino acid sequence, but their extracellular domains are quite divergent. Surprisingly, the soluble cleavage products of APLP1 and APLP2 also stimulated neurite outgrowth in our *in vitro *assay. In addition, we observed that knock down or knock out of APLP1 and APLP2 in neurons resulted in an increase in neurite length, similar to the situation in APP knock-out neurons, suggesting that APLP1 and APLP2 at the cell surface can also regulate neurite outgrowth. Moreover, we found that full length APLP1 and APLP2 also interact with Itgβ1. Taken together, these results suggest that APP family members all play a role in regulating neurite outgrowth.

## Conclusion

We recently showed that acute APP knock-down in embryonic rodent cortex dramatically retards the migration of cortical precursor cells into the cortical plate [29]. In addition, we show here that APP knock-down in the cortex results in neurons that display abnormally formed and disorganized neurites. Each of these effects was due to a cell-autonomous loss of APP, as the total number of cells receiving APP shRNA was very low, and full length APP at the cell surface appears to mediate these effects. We hypothesize that the effects of loss of APP on neuronal precursor migration and neurite outgrowth are functionally linked through the ability of APP to differentially interact with factors at or near the cell surface (for example, Itgβ1), and that APPs-α may regulate each of these processes.

A recent study showed that knock-in of APPs-α to the APP genomic locus can rescue many of the defects observed in APP knock out animals, including reductions in brain and body weight, defects in long term potentiation, and certain behavioral defects in spatial learning, exploratory activity, and locomotor activities [18]. These studies suggest that APPs-α can substitute for a deletion of APP to regulate these processes. However, we find that APPs-α does not rescue defects in neurite outgrowth observed upon deletion of APP. Rather, APPs-α application to wild-type neurons phenocopies APP deletion. Similarly, migration defects observed upon APP loss-of-function also are not rescued by APPs-α, and full length APP is required for cortical plate entry [29]. Thus, both full length APP and its principal cleavage product APPs-α can apparently act as independent factors with multiple, sometimes distinct activities. Whereas APPs-α has evolved its own independent functions [18], our results indicate that it also has the ability to regulate the function of full length APP in some developmental processes.

## Materials and methods

### Neurite outgrowth assays

Sprague Dawley rats and C57BL6 mice were obtained from Charles River (Boston, MA, USA) and Taconic (Albany, NY, USA). APP knock out and APLP2 knock out mice were obtained form Jackson Labs (Bar Harbor, ME, USA). Hippocampal and cortical primary neurons were plated and cultured as described with the following changes [17]. Briefly, E17 or E18 hippocampi or cortices were dissected, and dissociated with trypsin-EDTA. Cells were plated onto LabTek CC2 coated two-well chamber slides (Thermofisher; Rochester, NY, USA) in 'plating media' (Dulbecco's modified Eagle's medium + 5% fetal calf serum + penicillin/streptomycin). Then, 2–4 h after plating, media was changed to Neurobasal medium containing B27 supplement. After 3 DIV, neurons were fixed in 4% paraformaldehyde, washed with phosphate-buffered saline (PBS), blocked in 2% donkey serum plus 0.1% Triton X-100, and outgrowth was examined by immunostaining with anti-βIII-tubulin antibody (1:1,000; Millipore, Billerica, MA, USA). Neurite outgrowth was quantified by imaging neurons on a Zeiss Axioskop and digital images were acquired with an MC100 camera system. Neuronal processes were then analyzed using Axiovision LE 4.4 software. For each graph, data on neurite length were generated from at least two independent sets of neurons (for each condition) and greater than 200 cells were counted for each condition for each experiment (with the exception of neurons transfected with shRNA constructs, where 50–150 cells were counted per experiment). Significance was determined using Kruskal-Wallis nonparametric ANOVA and Dunn multiple comparisons tests. All analysis was performed blind.

For shRNA transfections, neurons were plated on CC2-coated two-chambered slides (LabTek). Three hours after plating, each well was transfected with 600 ng of shRNA and 400 ng of GFP plasmid using 2 μl of Lipofectamine. Three hours post-transfection, media was changed to Neurobasal with B27 supplement and analyzed as above.

For integrin antibody blocking experiments, primary neurons were plated as above, and Armenian hamster anti-CD29 (Itgβ1; 50 μl; BD Biosciences)or an isotype matched control antibody (50 μl; BD Biosciences) was added to culture media when media was changed to Neurobasal with B27 supplement. Neurite outgrowth was then analyzed as above.

CM from CHO cell lines stably expressing APPs-α (M Townsend and DJ Selkoe, unpublished or control CHO was collected and concentrated using a YM-3 Centricon filter (Thermofisher; Rochester, NY, USA). Similarly, CM from cell lines stably transfected with human APLP1 and APLP2 [10] was collected and concentrated. CM was added to neuronal cultures on the day of plating and analyzed as above.

For purification of human APPs-α (wild type), the APP ectodomain, spanning amino acids 1–612 (ending at the α-secretase cleavage site, APPs-α) and containing a carboxy-terminal six-histidine tag was cloned into a transfer vector for generation of recombinant baculovirus. APPs-α protein was expressed using a baculovirus expression system, which was performed at the Wistar Institute at the University of Pennsylvania. Briefly, recombinant baculovirus containing the APPs-α gene was generated and used to infect High Five cells (*Trichoplusia ni*). Cells were grown for 48 h to allow for protein expression and secretion before CM was harvested for purification. APPs-α was purified to homogeneity first through an ammonium sulfate precipitation (0–60%) cut and followed by an immobilized metal affinity chromatography (nickel chelating) purification (for binding: 20 mM sodium phosphate; 500 mM NaCl; 20 mM imidazole; wash: 80 mM imidazole; elute: 150 mM imidazole). Following purification, APPs-α was dialyzed against PBS. Purification of human APPs-α C117A was slightly modified due to the low expression levels of mutant APPs: rather than purification from CM, purification was from transfected High Five cell pellets lysed in RIPA buffer.

### *In utero *electroporation

Sprague Dawley rats (Charles River) were housed and cared for under the guidelines established by University of Connecticut and Harvard University's Institutional Animal Care and Use Committees (IACUC) in compliance with federal standards. Timed pregnant rats (E13 or E17) were anesthetized with Ketamine/Xylazine (100/10 mixture, 0.1 mg per g body weight, intraperitoneally). The uterine horns were exposed, and a lateral ventricle of each embryo injected with DNA constructs and Fast Green (2 mg/ml; Sigma; St. Louis, MO) via a microinjector (Picospritzer III, General Valve; Fairfield, NJ, USA) and pulled glass capillaries. shRNA constructs (1.0–1.5 μg/μl) was co-electroporated with 0.5 μg/μl pCAG-GFP. Electroporation was accomplished with a BTX square wave electroporator, at 75 V, for 50 ms on followed my 950 ms off for 5 pulses. The voltage was discharged across copper alloy oval plates placed on the uterine wall across the head of the embryo. Brains from postnatal pups were harvested in 4% paraformaldehyde by cardiac perfusion, sectioned coronally, and immunostained for Tbr1 (1:1,000; Millipore) and MAP2 (1:5,000; Abcam, Cambrige, MA, USA). Images were acquired using a Zeiss LSM 510 confocal microscope with Axiovert 100 M system.

For neurite outgrowth quantitative analysis, E14 rat cortices were electroporated as described above. Then, 48 h after electroporation, whole brains were dissected in Hanks Balanced Salt solution (Gibco Invitrogen; Carlsbad, CA, USA), and GFP positive regions of the cortex were dissected out. Regions of electroporation from at least five independent brains for each condition were dissected and pooled. These electroporated regions were then dissociated and plated on CC2-coated two-chamber slides. Three days after plating, cells were fixed, immunostained, and analyzed as described above.

### Immunostaining

Primary E18 wild-type neurons were plated and fixed six days later. Neurons were fixed in 4% paraformaldehyde, then incubated in blocking buffer (2% donkey serum; 0.005% Triton X-100 in PBS) for greater than 1 h. Sections were then incubated in primary antibody (for APP: 22C11; 1:500; Millipore; for APLP2: W2CT; 1:500 [10]) overnight at 4°C, followed by three washes in PBS. Sections were then incubated with Cy3- and Cy2-conjugated secondary antibodies (1:500; Jackson Immunoresearch; West Grove, PA, USA) for greater than 1 h followed by four PBS washes. Sections were mounted with glass cover slips using GelMount (Biomeda Plovdiv, Bulgaria). Images were acquired using a Zeiss LSM 510 confocal microscope with Axiovert 100 M system.

### Plasmid generation

APP shRNA generation and testing were described previously [29]. shRNA constructs were generated in the pENTR-U6 vector (Invitrogen)with the following sequences: APP shRNA-inactive, gctgacaagaaggccgttatc; APP shRNA-active, gcactaacttgcacgactatg; APLP1 shRNA2 (active), cagattaatgaggtgatgc; APLP2 shRNA2 (active), gccacatcgcattcttcaagc. shRNAs were tested by transient co-transfections (Lipofectamine, Invitrogen) in CHO cells of shRNA construct (or vector) and carboxy-terminally FLAG-tagged APLP1 or APLP2. Cells were lysed after 48 h in 1% NP-40 STEN buffer (150 mM sodium chloride, 50 mM Tris, 2 mM EDTA and 1.0% (v/v) Nonidet P-40). Lysates were electrophoresed on 10–20% Tricine gels (Invitrogen) and transferred to nitrocellulose. Western blotting was performed with anti-M2 FLAG (1:1,000; Sigma) and IRDye800-conjugated anti-mouse secondary antibody (1:10,000; Rockland Immunochemicals, Gilbertsville, PA, USA) and detected using the Licor detection system.

Carboxy-terminally tagged murine APP with the FLAG epitope and carboxy-terminally tagged mouse Itgβ1 with the FLAG epitope were cloned into the pCAGGs plasmid, which drives expression under the chicken β-actin promoter and the CMV enhancer. FLAG-tagged angiotensin converting enzyme cDNA was cloned previously as described [43].

### Immunoprecipitation and western blotting

CHO cells were transiently co-transfected using Lipofectamine 2000 (Invitrogen) in 10 cm dishes with murine Itgβ1 with a carboxy-terminal FLAG tag with either vector alone or with human APP of the 695 or 751 splice variants, APP harboring two point mutations in the NPXY domain of APP (APP-NATA), APPs-α, APLP1, or APLP2. Cells were lysed after 48 h in 1% NP-40 STEN buffer (150 mM sodium chloride, 50 mM Tris, 2 mM EDTA and 1.0% (v/v) Nonidet P-40). Lysates were immunoprecipitated with M2-agarose (Sigma). Immunoprecipitations then were electrophoresed on 10–20% Tricine gels (Invitrogen) and transferred to nitrocellulose. Western blotting was performed with anti-APP (C9; 1:1,000; Selkoe lab), anti-APP (22C11; 1:500; Millipore), anti- APLP1 (WINT; 1:500; gift of D Walsh), or APLP2 (W2CT; 1:500; gift of D Walsh) and IRDye800- or IRDye680-conjugated secondary antibodies (1:10,000; Rockland Immunochemicals) and detected using the Licor detection system.

Similarly, APP-FLAG and human Itgβ1 were co-transfected into CHO cells. After 48 h, CM from control CHO cells or APPs-α CHO cells was applied to transfected cells for 8–12 h. Cells were lysed as above, immunoprecipitated with M2-agarose, and western blots performed with anti-APP as above and anti-Itgβ1 (Millipore). Alternatively, APP and Itgβ1-FLAG were co-transfected as above and purified APPs-α applied to transfected cells for 8–12 h, lysed and immunoblotted as above.

For endogenous co-immunoprecipitations, lysates from E18 rat primary neurons, rat cortices, mouse brains, CHO cells, or transfected CHO cells were immunoprecipitated with anti-Itgβ1 (MAB 1997; Millipore), anti-APP (C9; Selkoe Lab), or anti-Tbr1 as a control (Abcam).

## Abbreviations

APP: β-Amyloid precursor protein; CHO: Chinese hamster ovary; CM: conditioned media; DIV: days *in vitro*; E: embryonic day; GFP: green fluorescent protein; Itgβ1: Integrin β1; PBS: phosphate-buffered saline; shRNA: small hairpin RNAs.

## Competing interests

The authors declare that they have no competing interests.

## Authors' contributions

TLY—P and DJS conceived of the design of the study, TLY—P coordinated and contributed to the execution of all experiments performed in this study, analysis and interpretation of the data, and drafted the manuscript. AC generated purified APPs-α, RC generated neuronal cultures and aided in generation of plasmids used in the study, CM participated in the analysis of neurite outgrowth assays. In addition to study design, DJS participated in the interpretation of the data and aided in writing of the manuscript. All authors approved the final manuscript.
